# POU2F2 regulates glycolytic reprogramming and glioblastoma progression via PDPK1-dependent activation of PI3K/AKT/mTOR pathway

**DOI:** 10.1038/s41419-021-03719-3

**Published:** 2021-04-30

**Authors:** Rui Yang, Mei Wang, Guanghui Zhang, Yanping Li, Lulin Wang, Hongjuan Cui

**Affiliations:** 1 Key Laboratory of Precision Oncology of Shandong Higher Education, Institute of Precision Medicine, Jining Medical University, Jining, China; 2 State Key Laboratory of Silkworm Genome Biology, Southwest University, Chongqing, China; 3 Key Laboratory of Basic Pharmacology of Ministry of Education and Joint International Research Laboratory of Ethnomedicine of Ministry of Education, Zunyi Medical University, Zunyi, China; 4 Cancer center, Medical Research Institute, Southwest University, Chongqing, China; 5 Key Laboratory of Molecular Pharmacology, Liaocheng People’s Hospital, Liaocheng, China

**Keywords:** CNS cancer, Cancer metabolism

## Abstract

The POU Class Homeobox 2 (POU2F2) is a member of POU transcription factors family, which involves in cell immune response by regulating B cell proliferation and differentiation genes. Recent studies have shown that POU2F2 acts as tumor-promoting roles in some cancers, but the underlying mechanism remains little known. Here, we identified that the highly expressed POU2F2 significantly correlated with poor prognosis of glioblastoma (GBM) patients. POU2F2 promoted cell proliferation and regulated glycolytic reprogramming. Mechanistically, the AKT/mTOR signaling pathway played important roles in the regulation of POU2F2-mediated aerobic glycolysis and cell growth. Furthermore, we demonstrated that POU2F2 activated the transcription of PDPK1 by directly binding to its promoter. Reconstituted the expression of PDPK1 in POU2F2-knockdown GBM cells reactivated AKT/mTOR pathway and recovered cell glycolysis and proliferation, whereas this effect was abolished by the PDPK1/AKT interaction inhibitor. In addition, we showed that POU2F2-PDPK1 axis promoted tumorigenesis by regulating glycolysis in vivo. In conclusion, our findings indicate that POU2F2 leads a metabolic shift towards aerobic glycolysis and promotes GBM progression in PDPK1-dependent activation of PI3K/AKT/mTOR pathway.

## Introduction

The POU Class Homeobox 2 (POU2F2) belongs to POU transcription factors family that employ POU-specific domain to binding to DNA and activating transcription^1,2^. POU2F2 is originally considered as a B-cell-specific transcription factor, which regulates B cell proliferation and differentiation by binding to immunoglobulin gene promoters^3,4^. Recent studies have shown that POU2F2 also expressed in some solid tumors and provided the prognosis of cancer patients, including clear cell renal cell carcinoma, gastric cancer, and pancreatic cancer^5–7^. POU2F2 functions as a bond to linking NF-κB and SLIT2/ROBO1 interaction network and promoting gastric cancer metastasis^8^. POU2F2 activates the transcription of NAMPT to promoting triple-negative breast cancer metastasis and growth^9^. Previous studies focused on the functions of POU2F2 in cell proliferation and metastasis, but the roles of POU2F2 in biological processes remains unknown. To confirm the possibility of POU2F2 as a therapeutic target for cancer, the role of POU2F2 in different cancers and mechanism of action of POU2F2 in biological processes in cancer cell needs further investigation. In this study, we explored the role of POU2F2 in GBM progression.

GBM is the most common and lethal form of gliomas, with about 90% primary tumor^10^. GBM is difficult to treat and prone to recurrence due to their ability to tolerate a complex stress environment. Therefore, the prognosis of GBM patients is very poor, with a median overall survival of ~14.6 months, and only about 6.8% of GBM patients survive 5 years^11,12^. Metabolic reprograming is one of the main reasons that GBM cells adapt to stressful environments and maintain malignant proliferation^13^. Particularly, abnormal glycolytic activity is the most common way of metabolism reprogramming in GBM cells, as opposed to oxidative phosphorylation in normal cells^14^. This shift provides carbon and nitrogen sources for lipid and DNA synthesis for GBM cells and maintains cellular redox homeostasis^15^. In addition, the metabolite of aerobic glycolysis can inhibit immune cell infiltration and suppresses the antitumor ability of immune cells^16^. The glycolytic reprogramming exists in multiple genotypes of GBM. Therefore, targeted therapy for glycolytic abnormalities is more successful in highly heterogeneous tumors, such as GBM, than targeted therapy for abnormal genes.

In our study, we characterized the roles of POU2F2 in patients’ prognosis and biological progression of GBM. POU2F2 expressed extremely high in GBM, and the highly expressed POU2F2 significantly correlated with poor prognosis of GBM patients. POU2F2 depletion suppressed cell proliferation and induced cell cycle arrest in GBM cells, and it acted as a key regulator of glycolytic reprogramming that shifted oxidative phosphorylation towards aerobic glycolysis. Mechanistically, POU2F2 activated PI3K/AKT/mTOR pathway to leading a metabolic shift towards aerobic glycolysis and promoting GBM progression in a PDPK1-dependent manner. Taken together, our results provided new insights into the biological roles of POU2F2 in pathological conditions to better understanding the underlying mechanism of glycolytic reprogramming in GBM, and identified that POU2F2 might be potential therapeutic target for GBM.

## Materials and methods

### Clinical samples

Paraffin-embedded GBM tissue microarrays (TMA) were purchased from Chaoying Biotechnology Co., Ltd. (Xian, China) and they were originally obtained from Tongxu County People’s Hospital of Henan Province. The GBM and normal tissues were collected for examination of mRNA levels of POU2F2 from Liaocheng People’s Hospital (Liaocheng, China), with written informed consent provided by the patients. This project was approved by the Institute Research Ethics Committee of Jining Medical University and Liaocheng People’s Hospital.

### Cell lines and cell culture

The human GBM cell lines (LN229, U87-MG, U-118, and U251), normal glial cell SVGP12, and human embryonic kidney (HEK) 293FT cells were obtained from ATCC. All of the cell lines were authenticated and confirmed to be mycoplasma-free before use. All cells were cultured as previously described^17^.

### Cell proliferation

MTT assay was employed to determining the ability of cell proliferation as described previously^17^.

### Plasmids, transfection, and infection

The hairpin oligonucleotides were synthesized in Beijing Genomics Institute (BGI, Beijing, China) and cloned into the pLKO.1 lentivirus vector. Primer sequences are listed in Supplementary Table S1. The full-length cDNA of human POU2F2 and PDPK1 gene were generated by PCR and constructed to pCDH-CMV-MCS-EF1-puro vector. PDPK1 promoter fragment and mutations of the consensus POU2F2-binding site were synthetic and constructed to pGL3-Basic. The plasmids transfection and lentiviruses infection were carried out as previously described^17^.

### BrdU staining

BrdU immunofluorescent staining was performed according to our previous study^17^.

### Flow cytometry analysis of cell cycle

For cell cycle assay, cells were stained with propidium iodide (PI) according to the manufacturer’s instructions (Roche, Basel, Switzerland).

### Western Blot

Cells were lysed with radioimmunoprecipitation buffer (Beyotime, Shanghai, China) to obtain total protein extraction. Protein concentrations were determined by bicinchoninic acid protein assay kit (Beyotime, Shanghai, China). Protein were separated on 10% gels by SDS-PAGE buffer and transferred to a polyvinylidene difluoride membrane (Millipore, Burlington, MA, USA). The membrane was incubated with a diluted primary antibody overnight at 4 °C. Then, the polyvinylidene fluoride membrane was incubated with the secondary antibody at room temperature for 2 h. Finally, the results were analysed with the ECL Prime Western blotting (WB) detection system (GE Healthcare). Each experiment was repeated at least three times. The primary antibodies are listed in Supplementary Table S2.

### Gene set enrichment assay (GSEA)

The transcriptome data (GSE79292) was downloaded from NCBI GEO. Data were normalized, significance determined by ANOVA, and fold change calculated with the Partek Genomics Suite. GSEA was performed with GSEA v4.1.0 for all differentially expressed genes.

### Glucose uptake and consumption

For glucose uptake assay, 2000 GBM cells were seeded in 96-well plates with glucose-free medium overnight, wash cell three times in PBS. Cells were preincubated with 100 uL KRPH buffer for 10 min. Added 2-DG to cells and incubate for 20 min at 37 °C. 2-DG6P levels were determined with microplate reader in kinetic mode at 37 °C according to the manufacturer’s instructions (Abcam, Cambridge, UK). For glucose consumption assay, cells were seeded in plates at 37 °C for 48 h, the glucose content was detected by using a Glucose Assay kit (Sigma, Burlington, MA, USA). Data were analyzed according to standard curve line and OD value. All experiments were performed at three times.

### Lactate and lactate dehydrogenase (LDH) assays

The lactate and LDH assays were performed according to the manufacturer’s instructions (Sigma, Burlington, MA, USA). Samples were treated as manufacturer’s instructions, OD value was analyzed by using a SYNERGY HTX multimode reader. All experiments were performed in triplicate.

### Extracellular acidification rate (ECAR) assay

For ECAR assay, 4 × 10^4^ cells were seeded into XF-96 cell culture well plates and cultured for 24 h. The medium was replaced by Seahorse DMEM containing 2 µM glutamine. Cells were cultured in a non-CO_2_ incubator at 37 °C for 60 min. The data were analyzed to cell numbers and plotted as ECAR (mpH/min) as a function of time by using XF-96p analyzer.

### Chromatin immunoprecipitation (ChIP) assay

Chromatin was isolated from 20 × 10^6^ GBM cells with or without POU2F2 knockdown. Chromatin was disrupted and immunoprecipitated with magnetic protein G dynabeads (Thermo Fisher Scientific, Inc.) using 2 μg of the specific antibody POU2F2 as described previously^18^. The quantitative PCR was employed to amounting the immunoprecipitated DNA for specific antibody. Each experiment was repeated three independent times. Primer sequences are listed in Supplementary Table S1.

### Luciferase assay

For luciferase assay, LN229 cells with or without POU2F2 knockdown were transfected with PDPK1 reporter or pGL3-basic luciferase reporter and Renilla luciferase plasmid for 24 h. Cells were lysed and assayed with Dual Luciferase Assay according to the manufacturer’s instructions (Promega, Madison, WI, USA). All the experiments were performed in triplicate.

### Xenograft models

The 5-week-old female nude mice (BALA/c, Beijing Laboratory Animal Research Center, Beijing, China) were used to establish intracranial and subcuticular xenograft tumor model. For intracranial implantation of GBM Cells in Mice, these mice were randomly divided into three groups (six/group), we injected 2 × 10^5^ LN229 cells (in 5 μL of DMEM per mouse), with or without regulation of POU2F2 knockdown or PDPK1 expression, intracranially into 5-week-old nude mice. The animals were killed 14 days after GBM cell injection. Tumor formation and phenotype were determined by histological analysis of hematoxylin and eosin (H&E) stained sections. Signs of disease progression were followed until the last mouse had died. Subcuticular xenograft tumor models were established as previously described^17^. Animal welfare and experimental procedures were carried out in accordance with the Guide for the Care and Use of Laboratory Animals (Ministry of Science and Technology of China, 2006).

### H&E and immunohistochemistry staining

The brains and tumor tissues were harvested, fixed in 4% (vol/vol) formaldehyde, and embedded in paraffin. Paraffin embedded tumor tissues were sectioned at 4 μm, deparaffinized, and rehydrated. H&E and immunohistochemistry staining were performed according to standard protocols. The antibodies were listed in Supplementary Table S2.

### Statistical analysis

All the statistical analyses were performed by GraphPad Prism version 7. Clinical patient and gene expression data were downloaded from R2: Genomics Analysis and Visualization Platform (https://hgserver1.amc.nl) and Chinese Glioma Genome Atlas (CGGA). The significance of different groups was estimated with two-tailed unpaired student’s *t*-test. For overall survival analysis, Kaplan–Meier with log-rank test was conducted. Quantitative data are expressed as the mean ± standard deviation. *P* < 0.05 was considered statistically significant. All observations were confirmed by at least three independent experiments.

## Results

### POU2F2 serves as a promising marker for prognosis of GBM patients

**Fig. 1:**
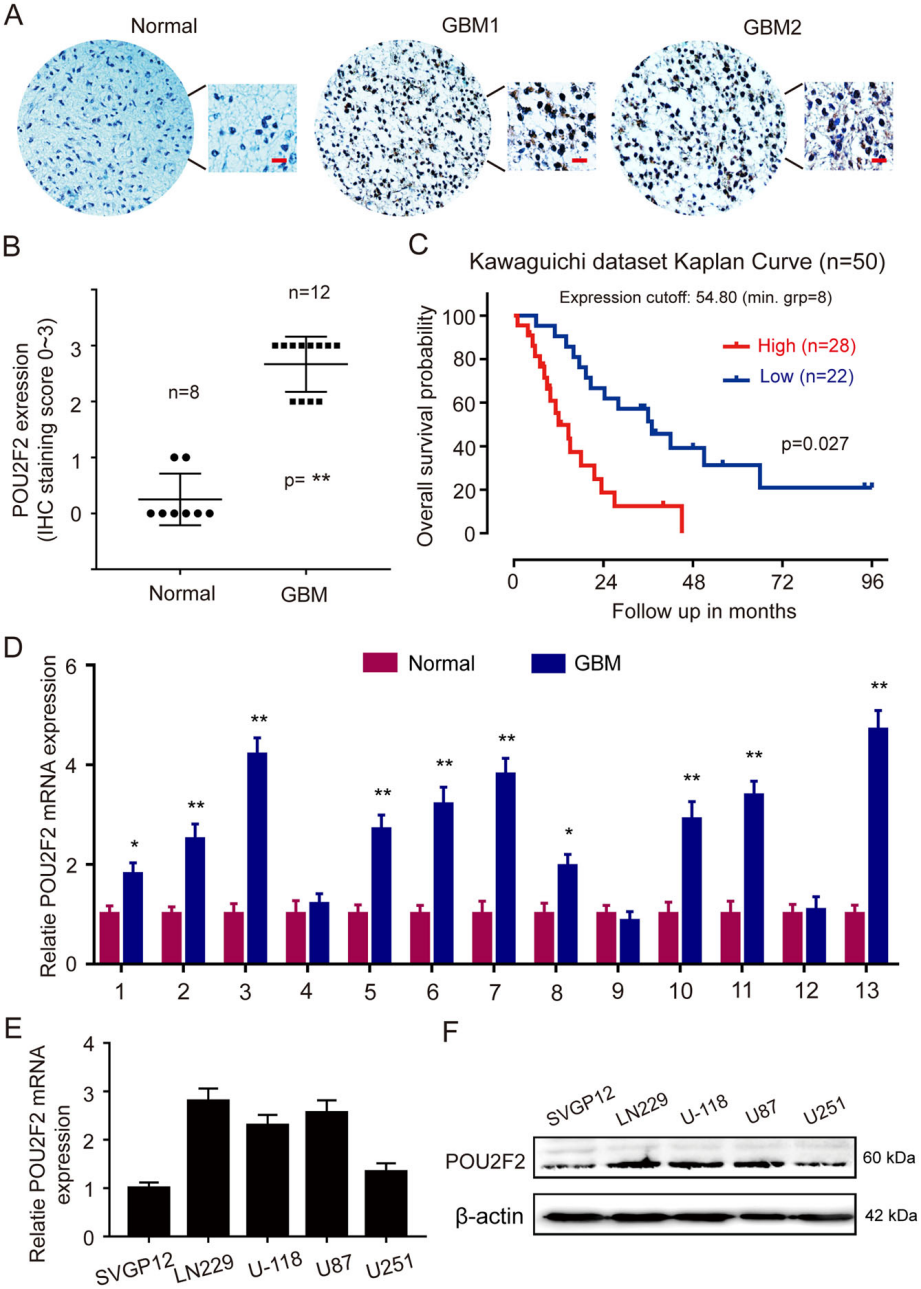
POU2F2 expression is upregulated and correlated with poor outcomes in GBM. **A** Immunohistochemical staining analysis of POU2F2 in normal brain tissues and 12 paired GBM tissue samples. **B** Immunohistochemistry analyses of POU2F2 expression levels in eight normal brain samples and 12 GBM samples. **C** Kaplan–Meier analysis of overall survival from the R2 database with the log rank test *P* value indicated. **D** Quantitative RT-PCR analysis of POU2F2 mRNA levels in 13 paired GBM and peritumoral normal tissues. **E**, **F** Quantitative RT-PCR and western blot analysis of POU2F2 expression in normal astrocytes (SVGP12) and GBM cell lines. All data were shown as the mean ± SD, *p < 0.05, **p < 0.01. All *p* values were based on analysis control versus treatment.

To understand the role of POU2F2 in GBM, we first demonstrated that POU2F2 expression levels correlate with GBM by performing immunohistochemical staining (IHC) using primary tissue microarray samples from GBM patients. POU2F2 expression was significantly increased in tumor samples from patients compared with normal tissues from individuals with no cancer (Fig. 1A, B). Besides this, different brain studies in Oncomine dataset showed that POU2F2 was significantly upregulated in GBM compared with normal tissues in Bredel and Lee dataset (Fig. S1A, B), but not in Rickman and Sun database (data not shown). We found that POU2F2 was higher expressed in GBM than it in low grade gliomas, including astrocytoma and oligodendroglioma (Fig. S1C, D). The analysis of CGGA patients’ data shown that POU2F2 expression was not correlated with age, gender, and IDH1 status (Fig. S1E). We evaluated the prognostic implication of POU2F2 expression in GBM patients based on the online data. The analysis of kawaguichi dataset demonstrated that GBM patients with high POU2F2 expression survived for a shorter time than those with low POU2F2 (Fig. 1C). We further examined the expression of POU2F2 in GBM based on our local patient cohort. We found that POU2F2 was significantly upregulated in GBM tissues compared with the paired peritumoral tissues (10/13) (Fig. 1D). We also detected the expression of POU2F2 in normal and GBM cells; POU2F2 was frequently higher expressed in GBM cells than those in normal glial cells (Fig. 1E, F). All these results suggested that POU2F2 expressed highly in GBM and served as a prognostic marker for GBM.

### POU2F2 is essential for cell growth in GBM

**Fig. 2:**
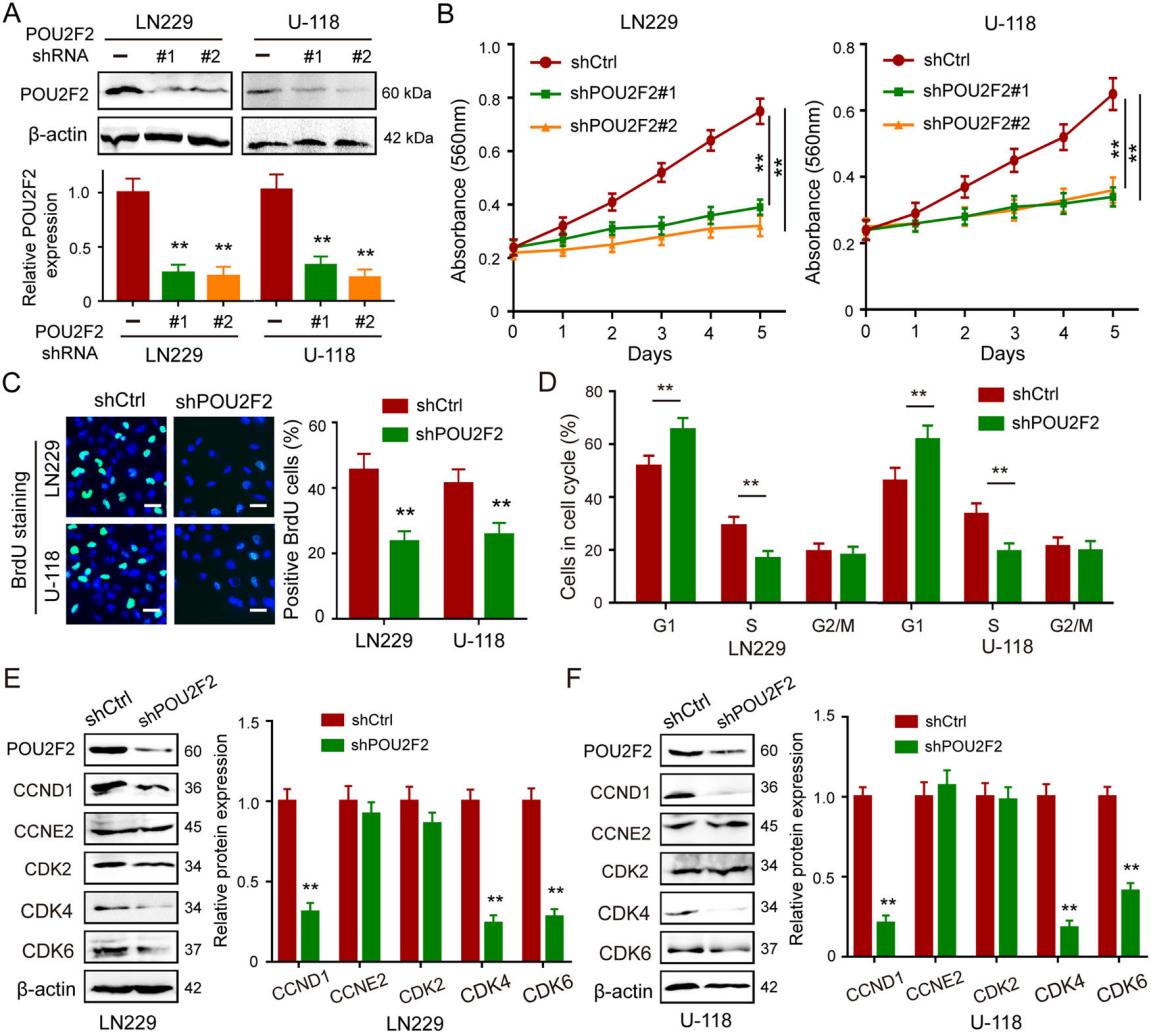
POU2F2 promotes cell proliferation by regulating cell cycle procession. **A** Western blot analyses of POU2F2 expressions in LN229 and U-118 cells transduced with POU2F2 shRNA clones or shRNA scramble. **B** The effect of POU2F2 on the proliferation of GBM cells was detected by MTT assays. **C** Image and quantification of GBM cells positive for BrdU staining. **D** The cell cycle analyses were performed in cells with shCtrl and shPOU2F2. The percentage of cells in the different phases were indicated. **E**, **F** Immunoblotting of POU2F2, CCND1, CCNE2, CDK2, CDK4, and CDK6 in GBM cells with or without POU2F2 silence. All data were shown as the mean ± SD, **p < 0.01.

We constructed POU2F2 knockdown cells to further investigating biological effects of POU2F2 in GBM (Fig. 2A). MTT assay was performed to examine cell viability, the results showed that POU2F2 silence obviously suppressed the proliferation ability of GBM cells (Fig. 2B), which was recovered by the reconstituted expression of POU2F2 (Fig. S2A). BrdU staining also demonstrated that POU2F2 knockdown induced a significant reduction in DNA synthesis (Fig. 2C). Soft-agar assay revealed that the ability of colony formation was inhibited in POU2F2 depletion cells compared with the control cells; this result further confirmed that POU2F2 is essential for GBM cell growth (Fig. S2B, C). The abnormal activity of cell proliferation is usually accompanied by changes in cell cycle progression, we next detected cell cycle of POU2F2 knockdown cells by using flow cytometry. Indeed, downregulation of POU2F2 induced cell cycle arrest at G1 phase (Fig. 2D). G1 phase-related proteins were detected to further verify above results. We found that the expressions of CCND1, CDK4, and CDK6, but not CCNE2 and CDK2, were significantly decreased after POU2F2 silence (Fig. 2E, F). These results demonstrated that POU2F2 regulated cell cycle progression and proliferation of GBM cells.

### POU2F2 plays important roles in regulation of glycolytic reprogramming

**Fig. 3:**
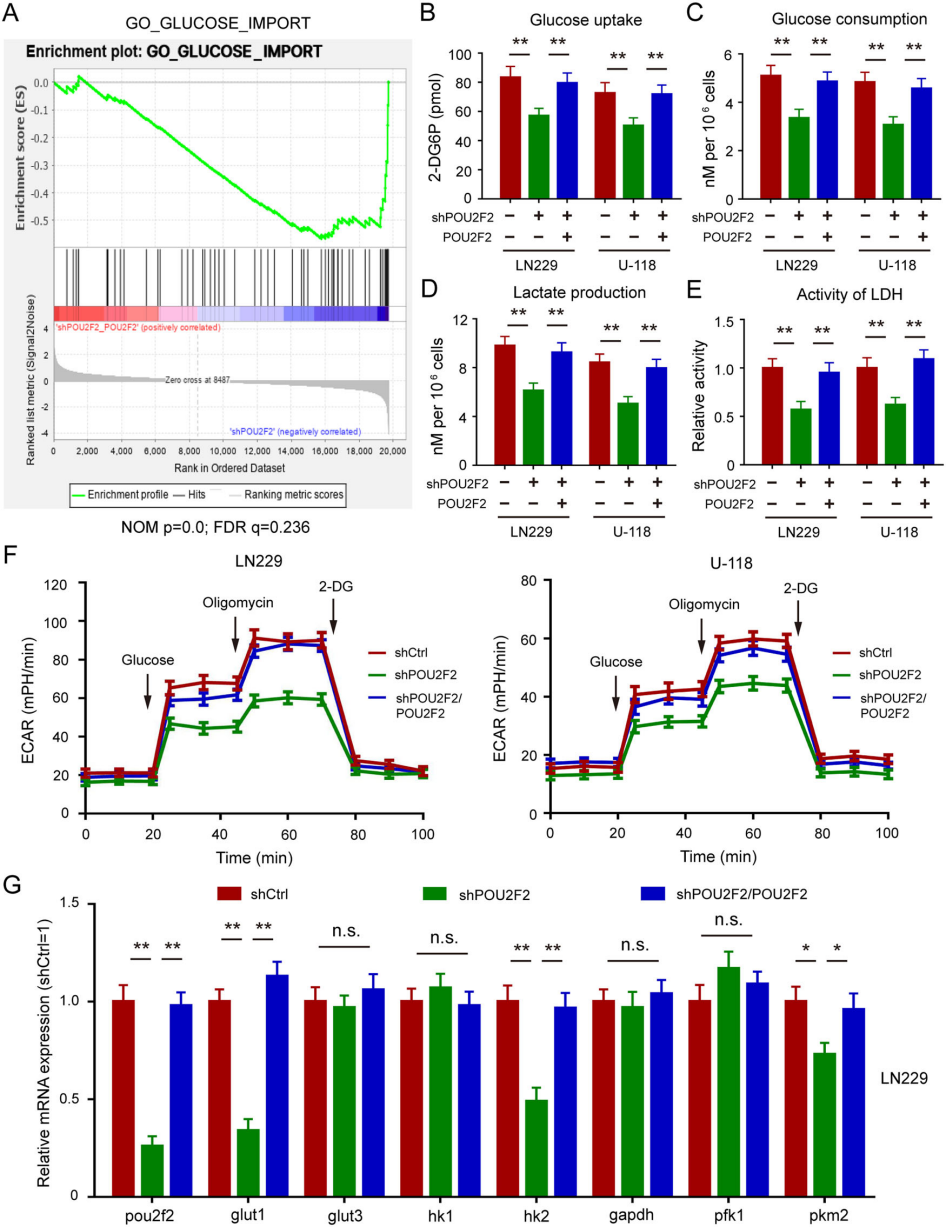
POU2F2 regulates glycolytic reprogramming in GBM cells. **A** GSEA showed significant enrichment of gene sets involved in the glucose import in B Cell lymphoma cells, shPOU2F2 compared with shPOU2F2/POU2F2. **B** Glucose uptake of GBM cells with or without POU2F2 silence and restoration of POU2F2. **C** Glucose consumption of GBM cells with or without POU2F2 silence and restoration of POU2F2. **D** Lactate production of GBM cells with or without POU2F2 silence and restoration of POU2F2. **E** Lactate dehydrogenase activities of GBM cells with or without POU2F2 silence and restoration of POU2F2. **F** Glycolytic flux changes of LN229 and U-118 cells. ECAR extracellular acidification rate. **G** Quantitative RT-PCR analysis of key glycolysis-associated enzymes levels in LN229 cells with or without POU2F2 silence and restoration of POU2F2. All data were shown as the mean ± SD, **p < 0.01.

To explore the underlying mechanism of promoted-proliferation induced by POU2F2, we analyzed the microarray data GSE79292^4^. Based on GSEA, we found that POU2F2 depletion led to lower expression of amount of glucose import genes in B Cell lymphoma cells (Fig. 3A). Then we detected glucose uptake of GBM cells with different POU2F2 condition. As shown in Fig. 3B, POU2F2 silencing remarkably inhibited glucose uptake of GBM cells, which was rescued by the reconstituted expression of POU2F2. We further explored the effects of POU2F2 in glucose metabolism of GBM cells. GO assays were performed to examine glucose consumption, the results showed that POU2F2 depletion significantly reduced glucose consumption of GBM cells (Fig. 3C). In lines with these results, lactate production and LDH activity were also decreased after POU2F2 knockdown (Fig. 3D, E).

Tumor cells preferentially initiate glycolysis to generating energy even when oxygen is sufficient, which is known as Warburg effect. Thus, we further detected whether POU2F2 affected glycolysis in GBM cells by assessing glycolytic flux using Seahorse XF analyzer. The results showed that glycolysis was attenuated in POU2F2 knockdown cells and was retrieved after POU2F2 restoration (Fig. 3F). To further determine whether POU2F2 lead GBM cells switch from oxidative phosphorylation to glycolysis, we treated cells with or without ATP synthase inhibitor oligomycin. ATP assays showed that POU2F2 silencing attenuated the production and inhibition rate of ATP (Fig. S3A, B). Next, we detected the expressions of key glycolysis-associated enzymes in POU2F2 depletion cells with or without restoration. We found that POU2F2 regulated glycolysis by modulating GLUT1, HK2, and PKM2 expression, but not GLUT3, HK1, GAPDH, and PFK levels (Figs. 3G and S3C). All these results demonstrated that POU2F2 remodeled glucose metabolism that lead a metabolic shift towards aerobic glycolysis.

### POU2F2 activates AKT/mTOR pathway to regulating glucose metabolism and cell growth

**Fig. 4:**
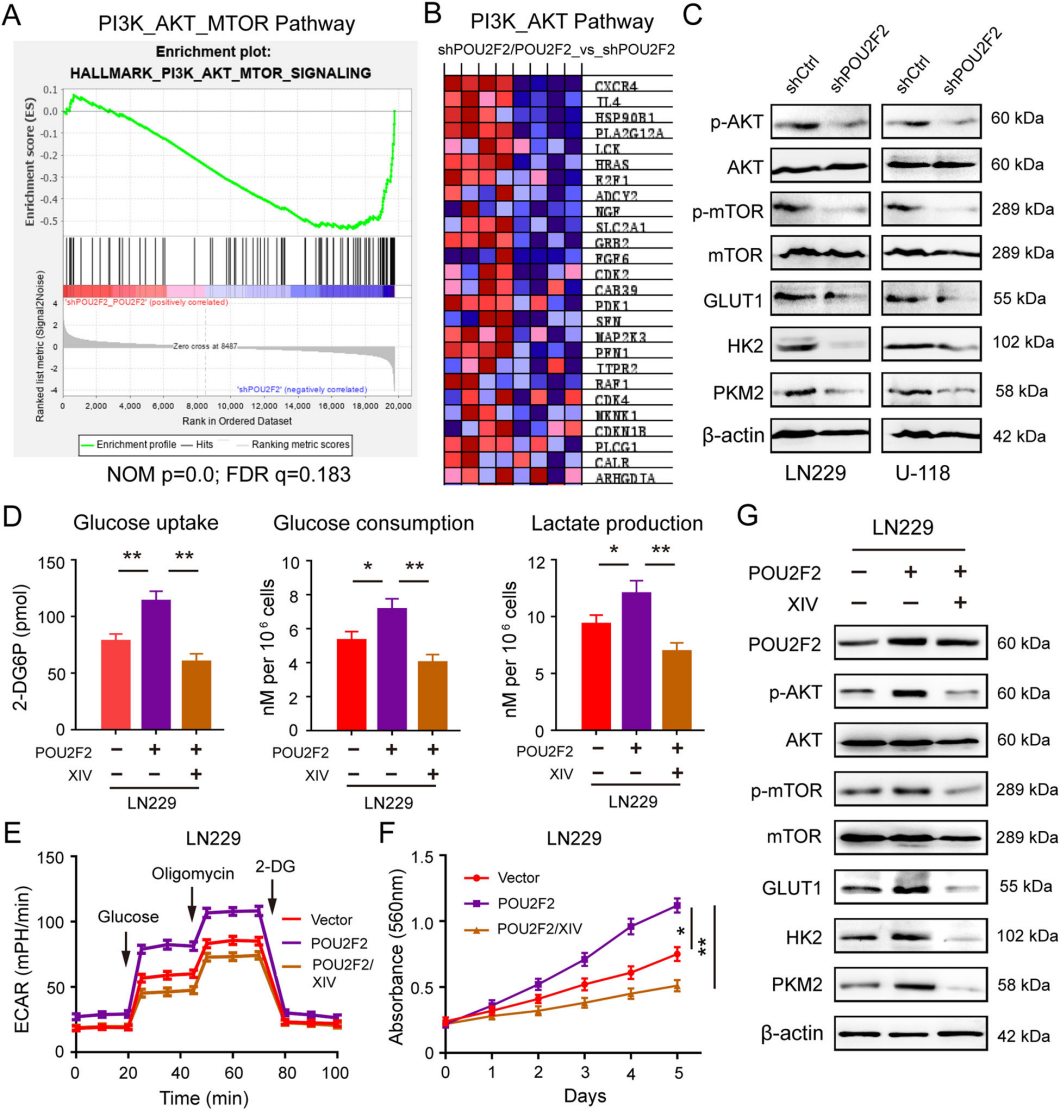
POU2F2 promotes aerobic glycolysis and cell growth by activating AKT/mTOR pathway. **A**, **B** GSEA showed significant enrichment of gene sets involved in the PI3K/AKT/mTOR pathway, with most of the genes being downregulated by POU2F2 depletion in B Cell lymphoma cells. **C** Immunoblotting of AKT, p-AKT (T308), mTOR, p-MTOR (S2448), GLUT1, HK2, and PKM2 in GBM cells with or without POU2F2 silence. **D** Glucose uptake, consumption, lactate production of LN229 cells with or without POU2F2 induction and XIV (20 μM). **E** Glycolytic flux changes of LN229 cells with or without POU2F2 induction and XIV. **F** Growth assays of LN229 cells with or without POU2F2 induction and XIV. **G** Immunoblotting of AKT, p-AKT, mTOR, p-MTOR, GLUT1, HK2, and PKM2 in LN229 cells with or without POU2F2 induction and XIV, β-actin levels were shown as loading control. All data were shown as the mean ± SD, *p < 0.05, **p < 0.01.

The GSEA of GSE7929 microarray data also showed that POU2F2-regulated genes were enriched in PI3K/AKT/mTOR pathway in B-lymphocyte cells (Fig. 4A, B). The activation of AKT/mTOR pathway is the central factor that remolds glycolysis by regulating some key glycolysis-associated enzymes, including GLUT1, HK2, and PKM2. In LN229 and U-118 cells, we found that downregulation of POU2F2 significantly inhibited the activation of AKT/mTOR pathway and its downstream GLUT1, HK2, and PKM2 (Fig. 4C). Thus, we hypothesized that POU2F2 might regulate glycolytic reprogramming through activating PI3K/AKT/mTOR pathway. We overexpressed POU2F2 in GBM cells and treated them with or without AKT inhibitor XIV, which could inhibit the phosphorylation of AKT^19^. According to the metabolite analysis, we found that overexpression of POU2F2 significantly promoted glucose uptake, consumption, and lactate production, and the effects were abrogated by inhibition of AKT signaling (Figs. 4D and S4A). In line with the results, ECAR assay showed that AKT inhibition blocked the metabolic shift towards aerobic glycolysis induced by POU2F2 overexpression (Figs. 4E and S4B). Meanwhile, AKT inhibitor XIV obviously reduced the increased levels of GLUT1, HK2, and PKM2 induced by POU2F2 (Fig. 4G). In addition, inhibition of AKT pathway abolished the increased proliferation of GBM cells induced by POU2F2 overexpression (Fig. 4F and S4C). These results indicated that POU2F2 promoted aerobic glycolysis and cell proliferation by regulating the activation of AKT/mTOR pathway.

### POU2F2 regulates AKT/mTOR pathway by directly activating the transcription of PDPK1

**Fig. 5:**
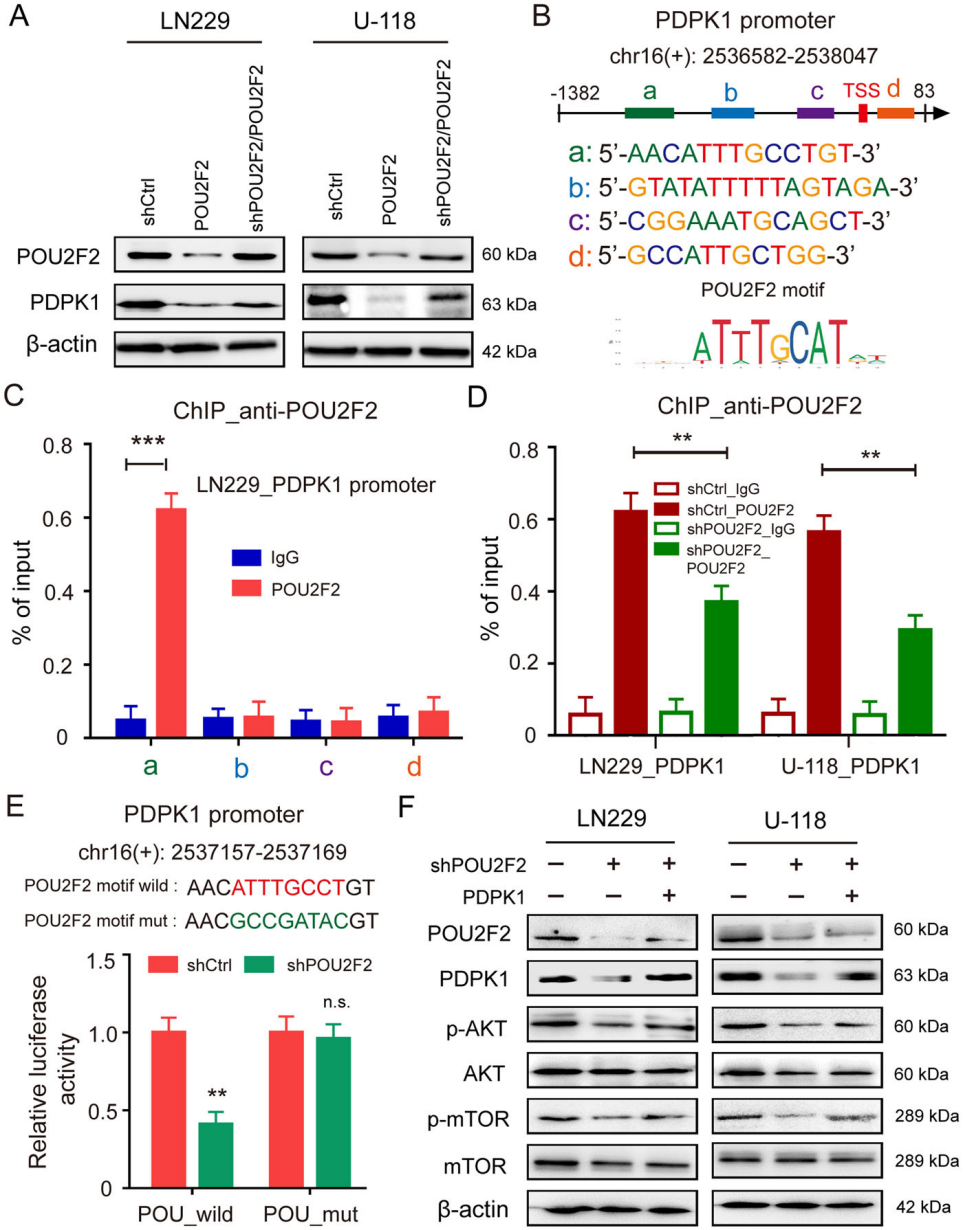
POU2F2 activates AKT/mTOR pathway by directly promoting the transcription of PDPK1. **A** Immunoblotting of POU2F2 and PDPK1 in GBM cells with or without POU2F2 silence and restoration of POU2F2. **B** Predicted sequences of POU2F2 motif in PDPK1 promoter by JASPAR. **C** ChIP-qPCR analysis of POU2F2 levels at different regions of PDPK1 promoter in LN229 cells. **D** ChIP-qPCR analysis of POU2F2 levels at PDPK1 promoter in LN229 and U-118 cells expressing shCtrl or shPOU2F2. **E** Luciferase promoter/reporter constructs containing the truncated wild PDPK1 promoter or POU2F2 motif mutant were co-transfected with a shPOU2F2 or with an empty vector (shCtrl) into HEK293FT cells, and luciferase activity was evaluated 24 h later. **F** Immunoblotting of POU2F2, PDPK1, AKT, p-AKT, mTOR, and p-MTOR in GBM cells with or without POU2F2 silence and restoration of POU2F2. All data were shown as the mean ± SD, **p < 0.01, ***p < 0.001.

To better understand the molecular mechanism of activation of AKT/mTOR pathway induced by POU2F2, we further analyzed the transcriptome data. We found that PDPK1 (also known as PDK1) was downregulated after POU2F2 knockdown in B Cell lymphoma cells (Fig. 4B), that could interact with AKT and phosphorylated it at T308. Our results confirmed that POU2F2 could regulate PDPK1 expression in GBM cells (Fig. 5A). Furthermore, analysis of the ChIP-seq dataset GSE32465 from ENCODE^20^ showed specific association of POU2F2 with the PDPK1 promoter, suggesting that it is a direct target gene of POU2F2 (Fig. S5A). We identified four potential binding sites of POU2F2 on PDPK1 promoter according to JASPAR dataset (Fig. 5B). ChIP-qPCR assay was performed to determine whether POU2F2 binds to PDPK1 promoter in GBM cells. Consistent with the ChIP-seq data, POU2F2 was highly enriched in the promoter region (−808 to −796) of PDPK1 (Figs. 5C and S5B). Besides this, knockdown of POU2F2 significantly decreased the enrichment of POU2F2 protein in PDPK1 promoter (Fig. 5D). We further verified whether POU2F2 directly regulated the transcription of PDPK1 by performing dual luciferase experiment. The different luciferase reporter vectors containing PDPK1 promoter with wild and mutant POU2F2 motif were transfected with shPOU2F2 or shCtrl in 293FT cells, and then the luciferase activities of these constructs were detected. The results showed that knockdown of POU2F2 significantly decreased the activity of PDPK1 promoter, whereas POU2F2 depletion failed to this effect on PDPK1 promoter with POU2F2 motif mutant (Fig. 5E). Then, we reconstituted the expression of PDPK1 in POU2F2-knockdown GBM cells. We found that the inhibited AKT/mTOR signaling pathway was reactivated by PDPK1 restoration (Fig. 5F). All these results suggested that POU2F2 activated AKT/mTOR pathway by directly promoting the transcription of PDPK1.

### POU2F2 promotes aerobic glycolysis and cell proliferation in a PDPK1-dependent activation of PI3K/AKT/mTOR pathway

**Fig. 6:**
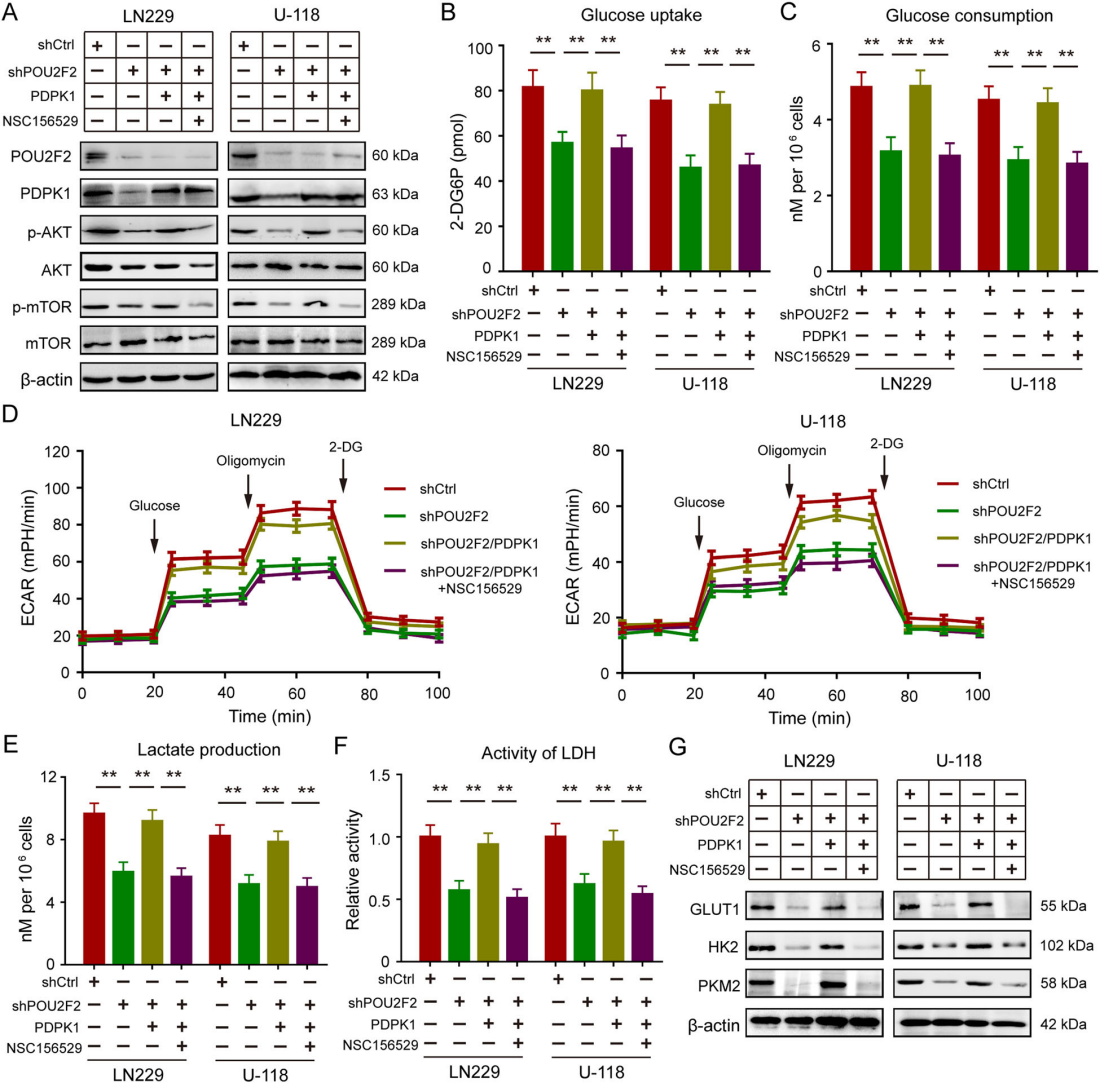
POU2F2 promotes aerobic glycolysis via PDPK1-dependent activation of PI3K/AKT/mTOR pathway. **A** Immunoblotting of POU2F2, PDPK1, AKT, p-AKT, mTOR, and p-MTOR in shCtrl, shPOU2F2, and shPOU2F2/PDPK1 cells with or without NSC156529 treatment (20 μM). **B** Glucose uptake in GBM cells with different treatment. **C** Glucose consumption in GBM cells with different treatment. **D** Glycolytic flux changes in GBM cells with different treatment. **E** Lactate production in GBM cells with different treatment. **F** Lactate dehydrogenase activity in GBM cells with different treatment. **G** Immunoblotting of GLUT1, HK2, and PKM2 in shCtrl, shPOU2F2, and shPOU2F2/PDPK1 cells with or without NSC156529 treatment. All data were shown as the mean ± SD, **p < 0.01.

To further verify whether PDPK1 mediated activation of AKT/mTOR pathway was involved in the promotion of aerobic glycolysis and cell proliferation induced by POU2F2, we used a PDPK1 inhibitor NSC156529 to inhibiting activation of AKT by abolishing the interaction between PDPK1 and AKT^21^. Here, we treated POU2F2 silence cells that reconstituted the expression of PDPK1 with NSC156529 and found that the recovered activation of AKT/mTOR pathway induced by reconstituted expression of PDPK1 was attenuated after NSC156529 treatment in POU2F2 depletion cells (Fig. 6A). Metabolite analyses showed that PDPK1 inhibitor NSC156529 obviously decreased glucose uptake and consumption of POU2F2 knockdown cells with restoration of PDPK1. The glycolytic stress flux tests were examined by ECAR assay, the results revealed that retrieved abilities of glycolysis induced by restored expression of PDPK1 were blocked after NSC156529 treatment in POU2F2 knockdown cells (Fig. 6D). In line with these results, lactate production and LDH activities were rescued by reconstituted expression of PDPK1 in POU2F2 silence cells, but these effects were abolished by NSC156529 treatment (Fig. 6E, F). Similarly, restoration of PDPK1 also increased the expression of key glycolysis-associated enzymes in POU2F2 knockdown cells, including GLUT1, HK2, and PKM2, the effect was abolished by NSC156529 treatment (Fig. 6G). In addition, the abilities of cell proliferation were rescued by reconstituted expression of PDPK1, and PDPK1-AKT interaction inhibitor NSC156529 abrogated this effect (Fig. S6A, B). All these results suggested that POU2F2 leaded metabolic shift towards aerobic glycolysis and promoted cell proliferation in PDPK1-dependent activation of PI3K/AKT/mTOR pathway.

### POU2F2 promotes GBM progression by regulating PDPK1/AKT/mTOR pathway

**Fig. 7:**
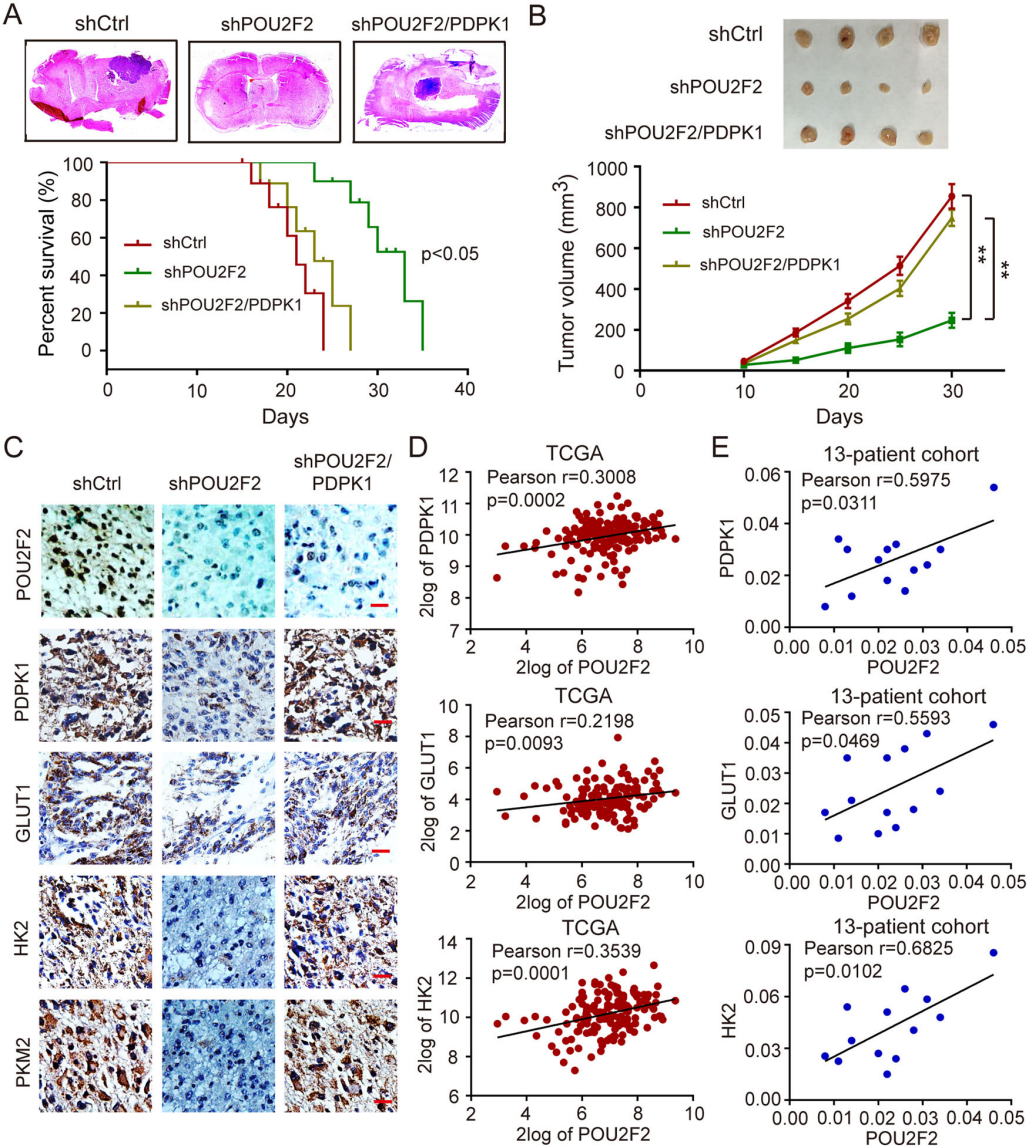
POU2F2-PDPK1 axis promotes brain tumorigenesis by regulating glycolysis. **A** Orthotopic tumorigenesis abilities of LN229 cells with depletion of POU2F2 and restoration of PDPK1 (top) and survival rates of mice (below). **B** Subcutaneous tumorigenesis abilities of LN229 cells depleted of POU2F2 and restoration of PDPK1. **C** Immunohistochemical staining analysis of POU2F2, PDPK1, GLUT1, HK2, and PKM2 in tumor tissues formed by GBM cells depleted of POU2F2 and restoration of PDPK1. **D** The correlation of POU2F2 expression with PDPK1, GLUT1, and HK2 in TCGA patients’ data. **E** The correlation of POU2F2 expression with PDPK1, GLUT1 and HK2 in our local patients’ data. All data were shown as the mean ± SD, **p < 0.01. All *p* values were based on analysis control versus treatment.

**Fig. 8:**
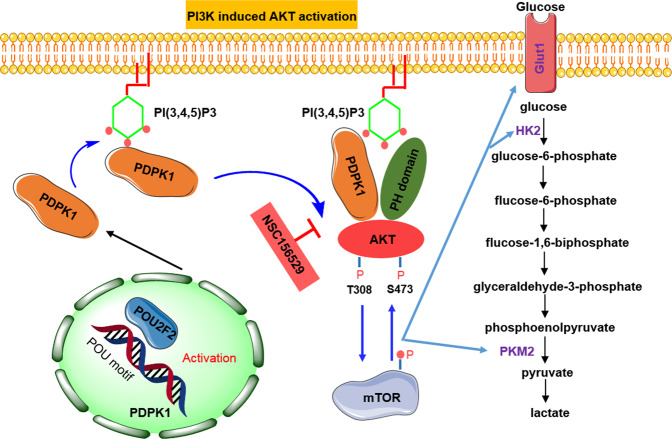
A mechanism of POU2F2 regulates glycolytic reprogramming and GBM progression. POU2F2 directly promoted the transcription of PDPK1, and then PDPK1 interacted with AKT and activated PI3K/AKT/mTOR pathway to increase the expression of GLUT1, HK2, and PKM2, leaded metabolic shift towards aerobic glycolysis and promoted GBM progression.

To investigate the role of POU2F2-PDPK1 axis in GBM tumorigenesis, we injected LN229 shCtrl, LN229 shPOU2F2, and LN229 shPOU2F2 cells with reconstituted expression of PDPK1 intracranially into nude mice. Knockdown of POU2F2 significantly suppressed the growth of brain tumors and increased the survival time of mice, and these effects were reversed by the recovered expression of PDPK1 (Fig. 7A). The subcutaneous xenograft models further confirmed that POU2F2 promoted tumor growth in a PDPK1 dependent manner (Fig. 7B). These results highlight the significance of POU2F2-PDPK1 axis in GBM development. The key glycolysis-associated enzymes in tumor tissues were detected by IHC. We found that the expressions of GLUT1, HK2, and PKM2 were obviously reduced in tumors formed by POU2F2 depletion cells, and these effects were rescued in tumors formed by shPOU2F2/PDPK1 cells (Fig. 7C). These results suggested that POU2F2-PDPK1 axis might promote GBM tumorigenesis by regulating aerobic glycolysis. In addition, we evaluated the correlation of POU2F2-PDPK1 axis and the key glycolysis-associated enzymes in GBM patients. The TCGA data showed that POU2F2 level was positively correlated with the expression of PDPK1, GLUT1 and HK2 in GBM patients (Fig. 7D). This clinical significant of correlation was further confirmed in our local patient specimens (Fig. 7E). Taken together, all of our results demonstrated that POU2F2 directly promoted the transcription of PDPK1, and then POU2F2 interacted with AKT, activated PI3K/AKT/mTOR pathway to increase the expression of GLUT1, HK2, and PKM2, leaded metabolic shift towards aerobic glycolysis, and promoted GBM progression (Fig. 8).

## Discussion

GBM is the most common tumor of the central nervous system with a poor prognosis^1^. It is difficult to treat and prone to recurrence due to their ability to tolerate a complex environment^13^. Glycolytic reprogramming is an important factor of GBM adaptation to stressful and various environments. GBM cells take up more glucose than normal glia cells to sustain proliferation^22^. Glucose enters into glycolysis rather than oxidative phosphorylation to provide carbon and nitrogen sources for lipid and DNA synthesis for GBM cells and maintains cellular redox homeostasis, which is known as the Warburg effect^23^. In addition, the metabolite of aerobic glycolysis can inhibit immune cell infiltration and suppresses the antitumor ability of immune cells^16,24^. Thus, targeted therapy for glycolytic abnormalities seems as a successful method in GBM. The activation of PI3K/AKT signaling is a main reason that GBM cells initiate glycolysis. Inhibition of AKT-driven glycolysis reduces proliferation and induces apoptosis in GBM cells^25^. In this study, we identified that POU2F2 might be an important activator of AKT pathway and promoted glycolysis in GBM.

POU2F2 is highly expressed in some solid tumors and provides the prognosis of cancer patients^5–7^. However, the role of POU2F2 in regulating tumor progression remains controversial. Here, we identified that POU2F2 was expressed highly in GBM and served as a prognostic marker for GBM. Then, by applying “gain and loss” strategy, we found that POU2F2 promoted GBM cell proliferation by regulating G1-S transit. GSEA of previous microarray data GSE7929 showed POU2F2 might be involved in glycolytic reprogramming in lymphoma cells. Depletion of POU2F2 significantly reduced glucose uptake, consumption, and lactate production in GBM. Besides this, POUF2 deficiency caused attenuated glycolysis and increased ATP production, suggesting that POU2F2 leaded metabolic shift towards aerobic glycolysis. We also investigated the effects of POU2F2 depletion on the key glycolytic enzymes. The expression levels of GLUT1, HK2, and PKM2 were significantly decreased in POU2F2 knockdown cells and GBM xenografts. The key glycolytic enzymes, including GLUT1, HK2, and PKM2 are highly expressed in GBM and play important roles in cell growth and tumor development^26–28^. We demonstrated that POU2F2 level was positively correlated with the expression of GLUT1 and HK2 in GBM patients. Our results suggested that POU2F2 promoted GBM progression by remodeling glucose metabolism.

Previous studies have demonstrated that POU2F2 might been involved in the activation of PI3K/AKT/mTOR signaling pathway^29^. We also confirmed that POU2F2 could activate PI3K/AKT/mTOR pathway in GBM cells. GBM is frequently accompanied with overexpression of EGFR and the activation of its downstream PI3K/AKT/mTOR pathway^30^. The PI3K/AKT signaling and their mammalian target of mTOR are key regulators of glycolytic reprogramming and cancer cell proliferation. Studies show that PI3K/AKT signaling is able to increase glucose transporter expression (GLUT1), enhance glucose uptake^31^. HIF-1α protein is stabilized by PI3K/AKT/mTOR pathway, and HIF-1α increases the transcription of some key glycolytic enzymes, including GLUT1, HK2, and PKM2^32,33^. Herein, we suppressed the PI3K/AKT/mTOR pathway in POU2F2-overexpresison cells by treating them with the AKT inhibitor XIV, which inhibits the phosphorylation of AKT at Ser 473^19^. The enhanced glycolysis and proliferation induced by POU2F2 overexpression were abrogated by inhibition of PI3K/AKT pathway. Our results indicated that POU2F2 likely promoted aerobic glycolysis and cell proliferation by regulating the activation of AKT/mTOR pathway.

The protein kinase-3-phosphoinositide-dependent kinase 1 (PDPK1) is essential for the activation of PI3K/AKT/mTOR pathway. PDPK1 interacts with AKT and phosphorylates it at Thr-308 dependent on PH domain. With the aid of PDPK2, the hydrophobic terminal of AKT is phosphorylated^34^. The double-phosphorylated Akt separates from the membrane, thus resulting in the activation of mTOR, and the activated mTOR in turn phosphorylates AKT at Ser-473^35^. Here, we demonstrated that POU2F2 promoted the transcription of PDPK1 by directly binding to its promoter site (ATTTGCCT). We also verified that POU2F2 activated the PI3K/AKT/mTOR pathway by promoting the expression of PDPK1 in GBM cells. Furthermore, reconstituted the expression of PDPK1 in POU2F2-knockdown GBM cells reactivated AKT/mTOR pathway and recovered cell glycolysis and proliferation, whereas this effect was abolished by the PDPK1/AKT interaction inhibitor. PDPK1 is highly expressed in GBM and play important roles in cell growth and tumor development^36^. We demonstrated that POU2F2 level was positively correlated with the expression of PDPK1 in GBM patients, suggesting the important roles of POU2F2-PDPK1 axis in the regulation of glycolytic reprogramming and GBM development.

In summary, we identify that POU2F2 is highly expressed in GBM and promotes GBM development. POU2F2 directly promotes the transcription of PDPK1, and then POU2F2 interacts with AKT and activates PI3K/AKT/mTOR pathway to increase the expression of GLUT1, HK2 and PKM2, leads metabolic shift towards aerobic glycolysis and promotes GBM progression. Our study provide new insights into the biological roles of POU2F2 in pathological conditions to better understanding the underlying mechanism of glycolytic reprogramming in GBM, and identify that POU2F2 is a potential therapeutic target for GBM.

## Supplementary information

Supplementary figure legends

Supplemental Table S1

Supplemental Table S2

Supplemental Figure S1

Supplemental Figure S2

Supplemental Figure S3

Supplemental Figure S4

Supplemental Figure S5

Supplemental Figure S6

## Data Availability

The transcriptome sequencing of POU2F2 in knockdown and restoration cells are deposited at the Gene Expression Omnibus database with the accession number GSE79292. The ChIP-seq of POU2F2 in GM12891 cells are deposited at the Gene Expression Omnibus database with the accession number GSE32465. The authors declare that all data supporting the findings of this study are available within the paper and its supplementary information files.
