# Supplementary material for: POU2F2 regulates glycolytic reprogramming and glioblastoma progression via PDPK1-dependent activation of PI3K/AKT/mTOR pathway
Source: Cell Death Dis. 2021 Apr 30;12(5):433. doi: 10.1038/s41419-021-03719-3 (PMC8087798; doi:10.1038/s41419-021-03719-3)
Supplement: Supplementary file 1 — Supplementary figure legends [file 41419_2021_3719_MOESM1_ESM.docx]

**Supplementary Methods**

**Soft agar assay**

In total, 2 × 10^3^ cells were mixed with 0.3% Noble agar in growth medium and plated into six-well plates containing a solidified bottom layer (0.6% Noble agar in growth medium). The colonies were photographed after 14 to 21 days and recorded.

**Measurement of mitochondrial ATP levels**

Mitochondrial ATP levels were determined using isolated mitochondria suspended in 0.5 mL BES buffer (75% [v/v] EtOH, 10 mM (NH4)_2_SO_4_). Samples were incubated at 90°C for 3 min followed by centrifugation for 20 min at 4°C at 16000 g. The supernatant was diluted 20 times in 20 mM Tris (pH 8). 150 μL of the diluted sample were transferred to a 96-well plate and incubated for 5 min at RT. 50 μL substrate solution (ATP detection kit, Abcam113849) were added to the samples and incubated for 15 min in the dark. Luminescence was measured for 10 s using a plate reader. Substrate solution without mitochondria was used as blank.

**Supplementary Figure legends**

**Fig. S1 POU2F2 is highly expressed in GBM. (A)** Studies from the Bredel brain dataset presented the increase in POU2F2 mRNA in GBM samples. **(B)** Studies from the Lee brain dataset presented the increase in POU2F2 mRNA in GBM samples. **(C)** Analysis of Kotliarov brain dataset shown the increase in POU2F2 expression in GBM samples. **(D)** Analysis of TCGA dataset shown the increase in POU2F2 expression in GBM samples. **(E)** The correlation between POU2F2 expression and age, gender and IDH1 status in CGGA dataset. All data were shown as the mean ± SD, *p < 0.05.

**Fig. S2 POU2F2 is** **essential for GBM cell growth. (A)** MTT assays showed proliferation abilities of GBM cell with POU2F2 depletion and restoration. **(B, C)** The effects of POU2F2 on the colony formation in POU2F2-knockdown GBM cells. All data were shown as the mean ± SD, **p < 0.01.

**Fig. S3 POU2F2 leads GBM cells switch from oxidative phosphorylation to glycolysis.** **(A)** ATP production of GBM cells with or without POU2F2 silence and restoration of POU2F2. **(B)** ATP inhibition induced by oligomycin in GBM cells with or without POU2F2 silence and restoration of POU2F2. **(C)** Quantitative RT-PCR analysis of key glycolysis-associated enzymes levels in GBM cells cells with or without POU2F2 silence and restoration of POU2F2. All data were shown as the mean ± SD, *p < 0.05, **p < 0.01.

**Fig. S4 POU2F2 promotes aerobic glycolysis and cell growth by activating AKT/mTOR pathway. (A)** Glucose uptake, consumption, lactate production of U-118 cells with or without POU2F2 induction and XIV. **(B)** Glycolytic flux changes of U-118 cells with or without POU2F2 induction and XIV. **(C)** Growth assays of U-118 cells with or without POU2F2 induction and XIV. All data were shown as the mean ± SD, *p < 0.05, **p < 0.01.

**Fig. S5 POU2F2 binds to the PDPK1 promoter region.** **(A)** ChIP-seq tag profiles for POU2F2 levels at the PDPK1 promoter in GM12891 cells. The ChIP-seq dataset GSE32465 was used for analysis. **(B)** ChIP-qPCR analysis of POU2F2 levels at different regions of PDPK1 promoter in U-118 cells. All data were shown as the mean ± SD, ***p < 0.001.

**Fig. S6 POU2F2 promotes cell proliferation via PDPK1-dependent activation of PI3K/AKT/mTOR pathway. (A, B)** The growth curve of shCtrl, shPOU2F2, shPOU2F2/PDPK1 GBM cells with or without NSC156529 treatment. All data were shown as the mean ± SD, **p < 0.01.
