# Supplementary material for: POU2F2 regulates glycolytic reprogramming and glioblastoma progression via PDPK1-dependent activation of PI3K/AKT/mTOR pathway
Source: Cell Death Dis. 2021 Apr 30;12(5):433. doi: 10.1038/s41419-021-03719-3 (PMC8087798; doi:10.1038/s41419-021-03719-3)
Supplement: Supplementary file 2 — Supplemental Table S1 [file 41419_2021_3719_MOESM2_ESM.docx]

**Table S1 Primers and shRNA sequences used in this study**

**Primers for qRT-PCR**

| Primer name 5′-3 | 5′-3′ |
| --- | --- |
| POU2F2-F  POU2F2-R  GLUT1-F | GGCCCAACTCATGTTGACG  TGCTGAGGTAGCTGGAATAGATT  GAGGTAGTCAGACGGAAGCTA |
| GLUT1-R | GAGGGGAACATGCAGTCATTT |
| GLUT4-F  GLUT4-R | TGGGCGGCATGATTTCCTC  GCCAGGACATTGTTGACCAG |
| HK1-F  HK1-R  HK2-F  HK2-R  GAPDH-F  GAPDH-R  PFK1-F  PFK1-R  PKM2-F  PKM2-R  PDPK1-F  PDPK1-R | GCTCTCCGATGAAACTCTCATAG GGACCTTACGAATGTTGGCAA  TTGACCAGGAGATTGACATGGG CAACCGCATCAGGACCTCA  ACAACTTTGGTATCGTGGAAGG GCCATCACGCCACAGTTTC  GCATGGGTATCTACGTGGGG CTCTGCGATGTTTGAGCCTC  ATAACGCCTACATGGAAAAGTGT TAAGCCCATCATCCACGTAGA  TTCCGAGCTGGAAACGAGTAT GGTCTCTTGCCTTAGGGAAGAA |
| β-actin -F | CATGTACGTTGCTATCCAGGC |
| β-actin -R | CTCCTTAATGTCACGCACGAT |

**Primers for ChIP assay**

| Primer name 5′-3 | 5′-3′ |
| --- | --- |
| PDPK1-a-F  PDPK1-a-R  PDPK1-b-F  PDPK1-b-R  PDPK1-c-F  PDPK1-c-R  PDPK1-d-F  PDPK1-d-R | CACTTTGGGAGGCGGAGAC  GTTGAAGCGATTCTCCTGC  GACATTAACAACTACAAGGCGCC  GGAAATCTGGGCTATCAAGTCA  TTCCGGGTTCGAGCACAG  TCCAGCTGCATTTCCGGGTT  AACCCGGAAATGCAGCTGGA  GAAGCGGAGCCCCAGCAAT |
| GAPDH-F | GGTAGGGAGTTCGAGACCAG |
| GAPDH-R | TCAACGCAGTTCAGTTAGGC |

**Primers for shRNA**

| Target Sequence | 5′-3′ |
| --- | --- |
| ShPOU2F21#1-F | CCGGTCACTGCTACGACGCCAAATACTCGAGTATTTGGCGTCGTAGCAGTGATTTTTG |
| ShPOU2F2#1-R | CCGGTCACTGCTACGACGCCAAATACTCGAGTATTTGGCGTCGTAGCAGTGATTTTTG |
| ShPOU2F2#2-F | CCGGGCTACCGACACCAAATCTATTCTCGAGAATAGATTTGGTGTCGGTAGCTTTTTG |
| ShPOU2F2#2-R | AATTCAAAAAGCTACCGACACCAAATCTATTCTCGAGAATAGATTTGGTGTCGGTAGC |
