# Supplementary material for: POU2F2 regulates glycolytic reprogramming and glioblastoma progression via PDPK1-dependent activation of PI3K/AKT/mTOR pathway
Source: Cell Death Dis. 2021 Apr 30;12(5):433. doi: 10.1038/s41419-021-03719-3 (PMC8087798; doi:10.1038/s41419-021-03719-3)
Supplement: Supplementary file 3 — Supplemental Table S2 [file 41419_2021_3719_MOESM3_ESM.docx]

**Table S2: Reagents and antibodies**

| **Reagents** | **Catalogue NO.** | **Company** |
| --- | --- | --- |
| XIV | CAS 1191951-57-1 | Sigma (Beverly, MA) |
| NSC156529 | SML1580 | Sigma (Beverly, MA) |
| GO Kit | GAGO20 | Sigma (Beverly, MA) |
| Lactate Kit | MAK064 | Sigma (Beverly, MA) |
| **Antibodies** | **Catalogue NO.** | **Company** |
| POU2F2 | sc-56822 X | Santa Cruz Biotechnology (Santa Cruz, CA) |
| CCND1 | 2922 | Cell Signaling Technology (Beverly, MA) |
| CCNE2 | 4132 | Cell Signaling Technology (Beverly, MA) |
| CDK2 | 2546 | Cell Signaling Technology (Beverly, MA) |
| CDK4 | 12790 | Cell Signaling Technology (Beverly, MA) |
| CDK6 | 3136 | Cell Signaling Technology (Beverly, MA) |
| p-AKT T308 | 13038 | Cell Signaling Technology (Beverly, MA) |
| AKT | 4685 | Cell Signaling Technology (Beverly, MA) |
| p-mTOR S2448 | 5536 | Cell Signaling Technology (Beverly, MA) |
| mTOR | 2972 | Cell Signaling Technology (Beverly, MA) |
| β-actin | ab8226 | Abcam (Cambridge, UK) |
| PDPK1 | ab52893 | Abcam (Cambridge, UK) |
| Glut1 | ab33780 | Abcam (Cambridge, UK) |
| HK2 | ab209847 | Abcam (Cambridge, UK) |
| PKM2 | ab137852 | Abcam (Cambridge, UK) |
