# Supplementary figures and images for: POU2F2 regulates glycolytic reprogramming and glioblastoma progression via PDPK1-dependent activation of PI3K/AKT/mTOR pathway
Source: Cell Death Dis. 2021 Apr 30;12(5):433. doi: 10.1038/s41419-021-03719-3 (PMC8087798; doi:10.1038/s41419-021-03719-3)

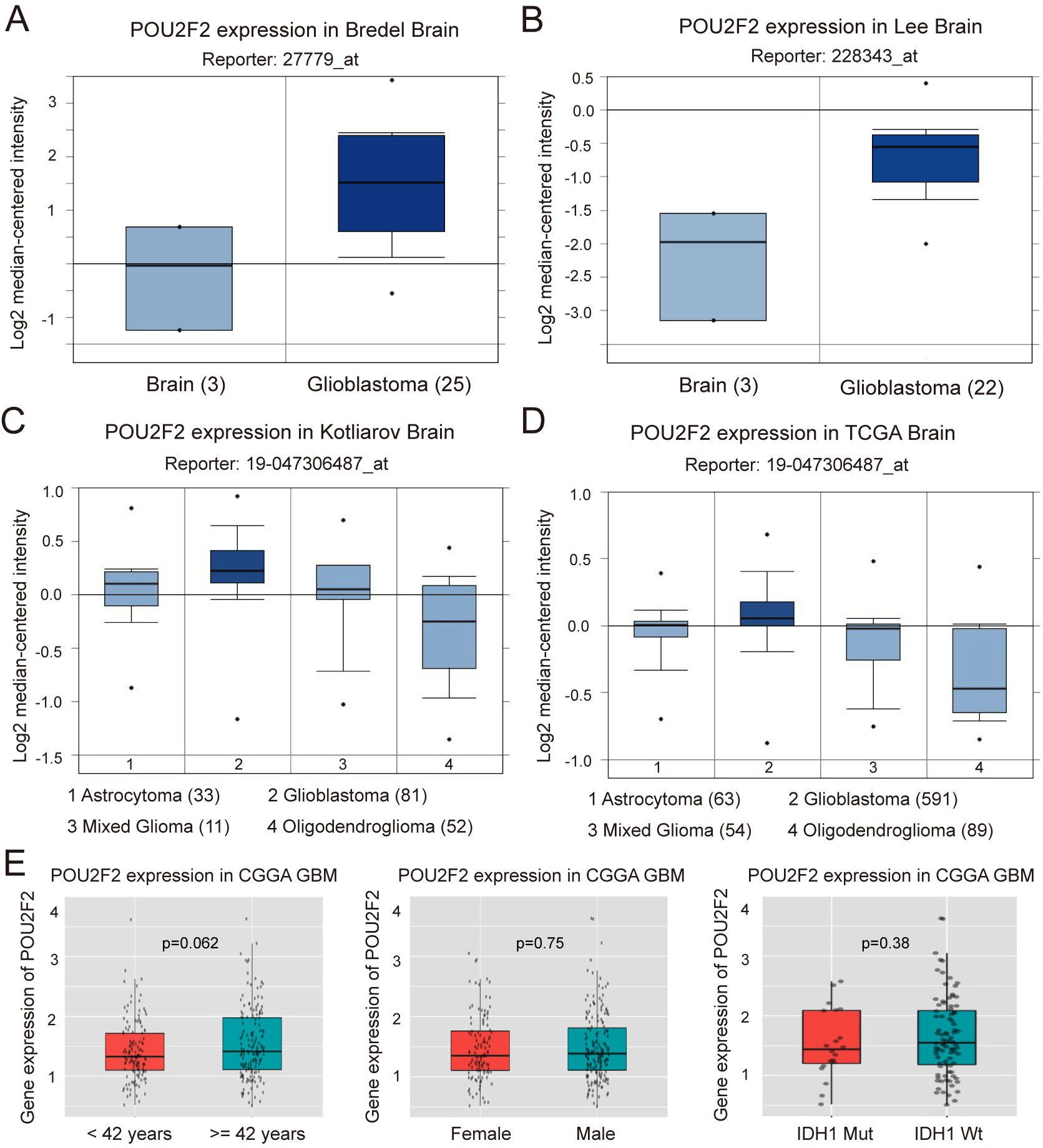

Supplement: Supplementary file 4 — Supplemental Figure S1 [file 41419_2021_3719_MOESM4_ESM.tif]

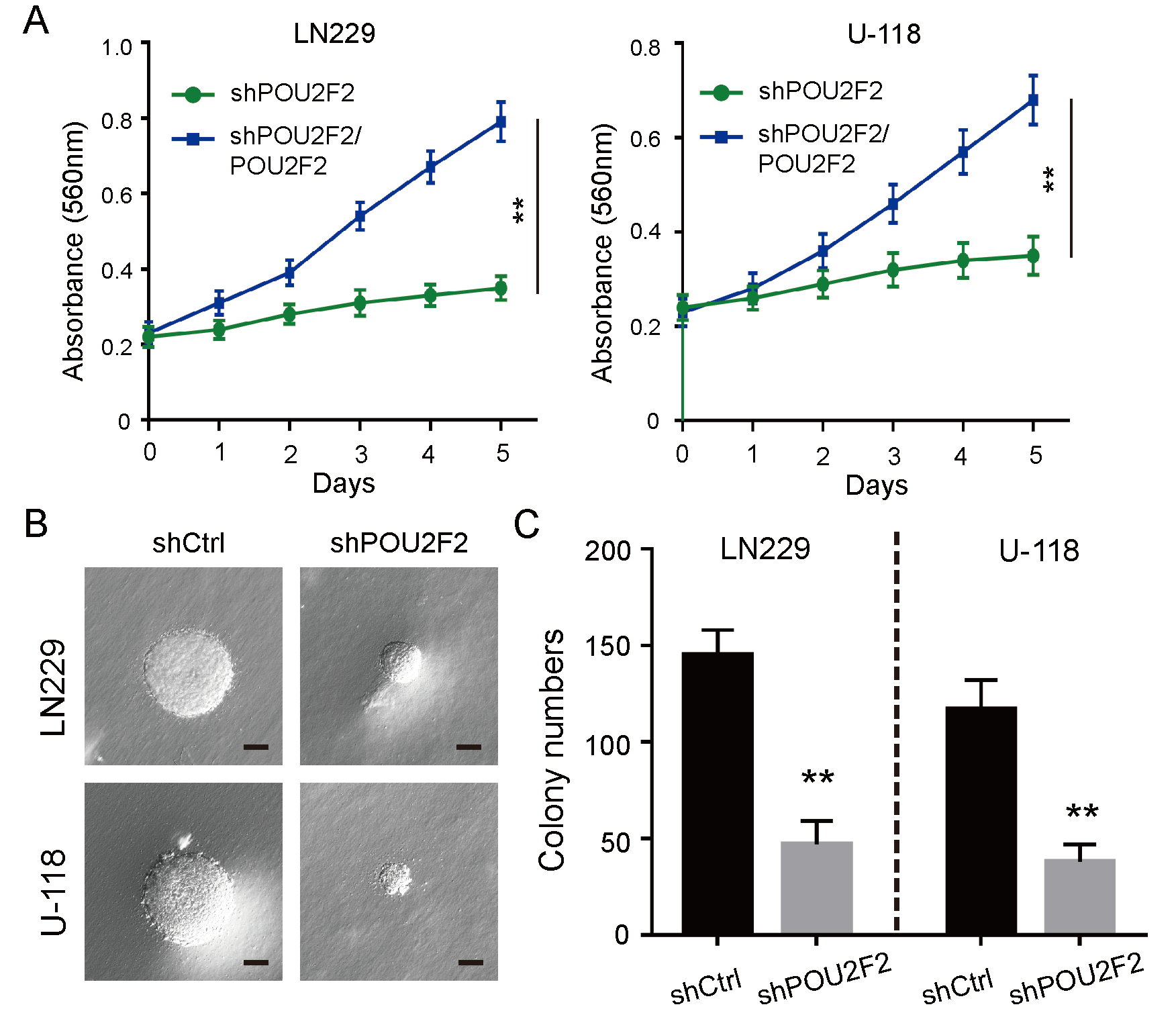

Supplement: Supplementary file 5 — Supplemental Figure S2 [file 41419_2021_3719_MOESM5_ESM.tif]

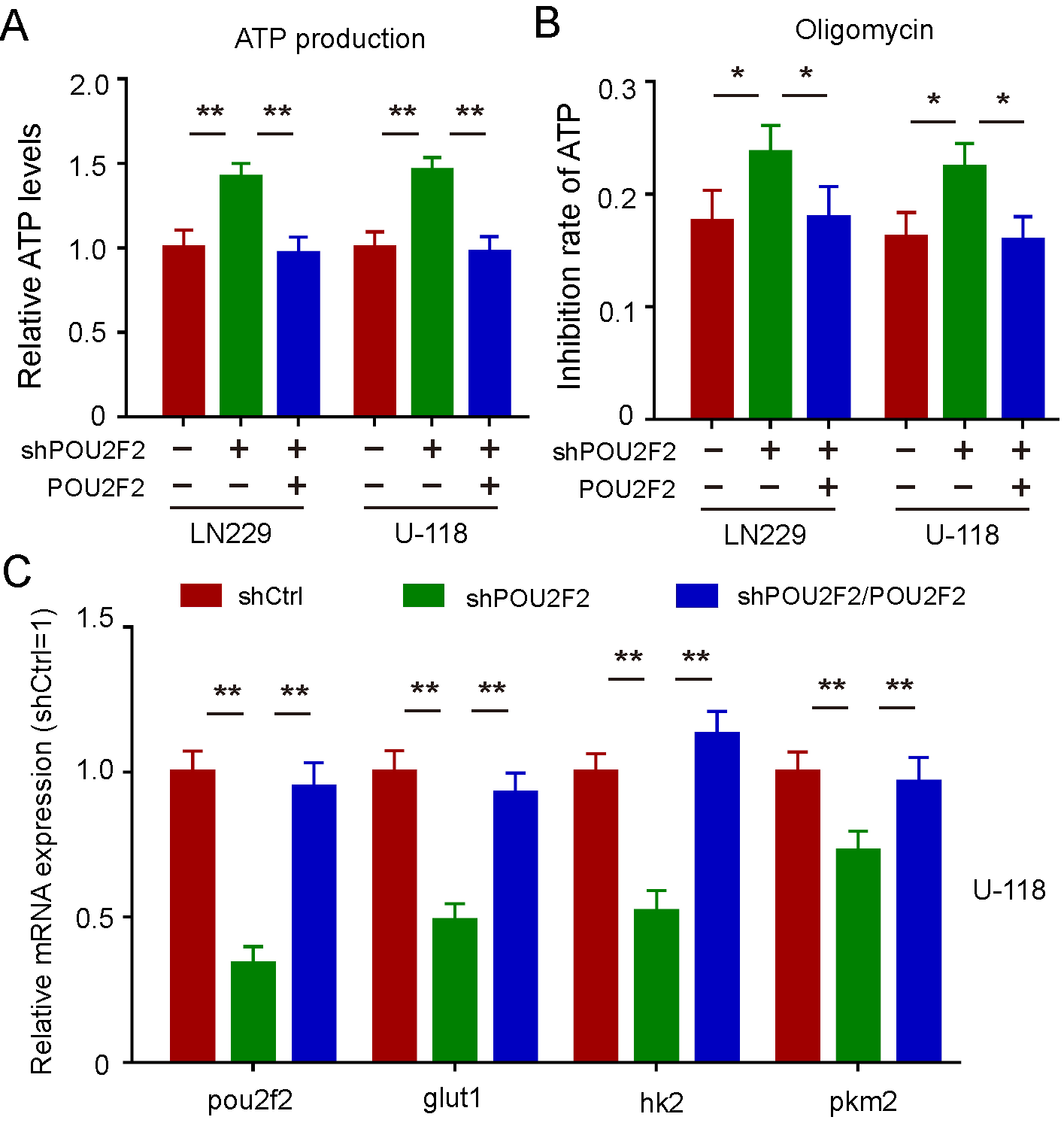

Supplement: Supplementary file 6 — Supplemental Figure S3 [file 41419_2021_3719_MOESM6_ESM.tif]

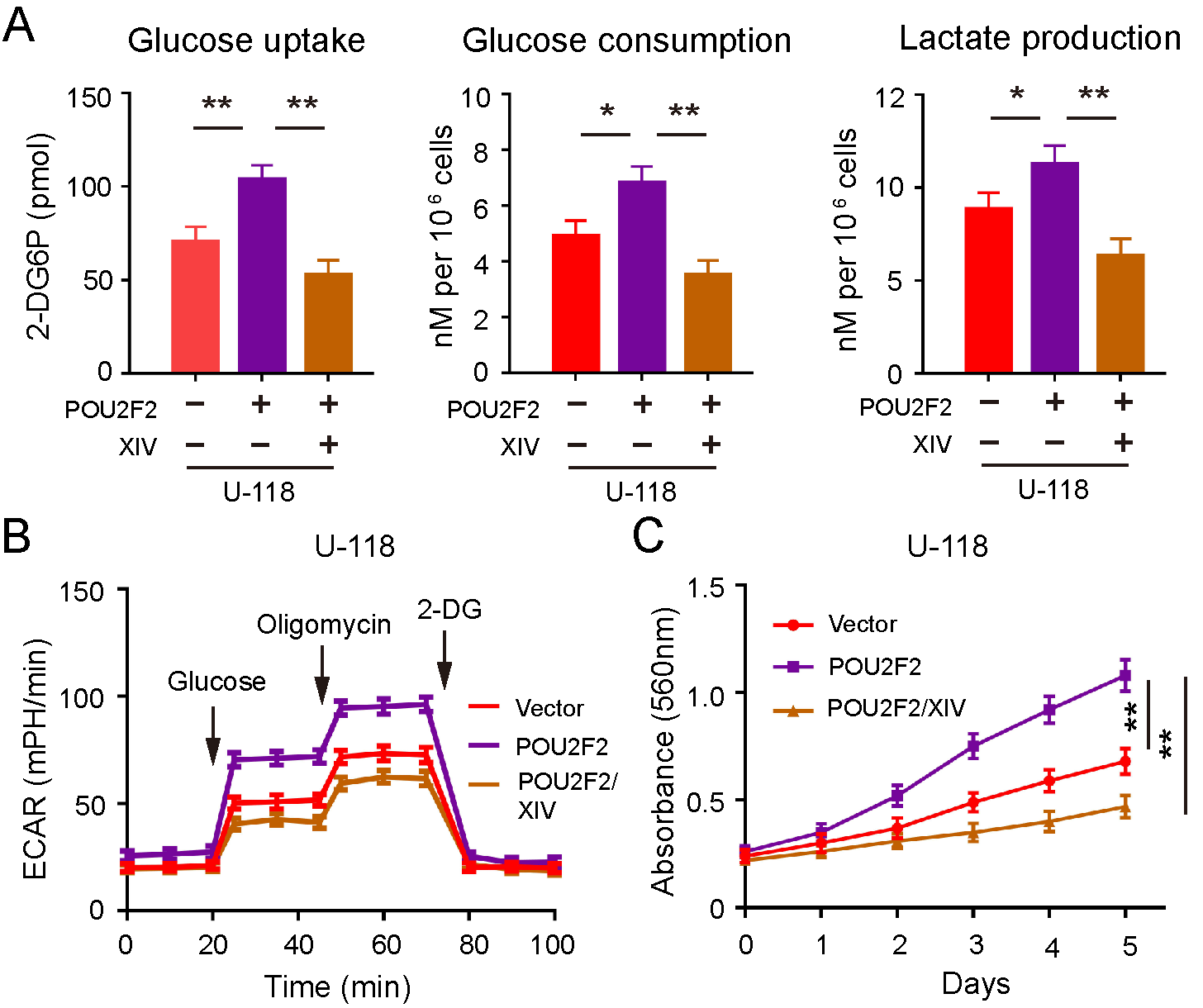

Supplement: Supplementary file 7 — Supplemental Figure S4 [file 41419_2021_3719_MOESM7_ESM.tif]

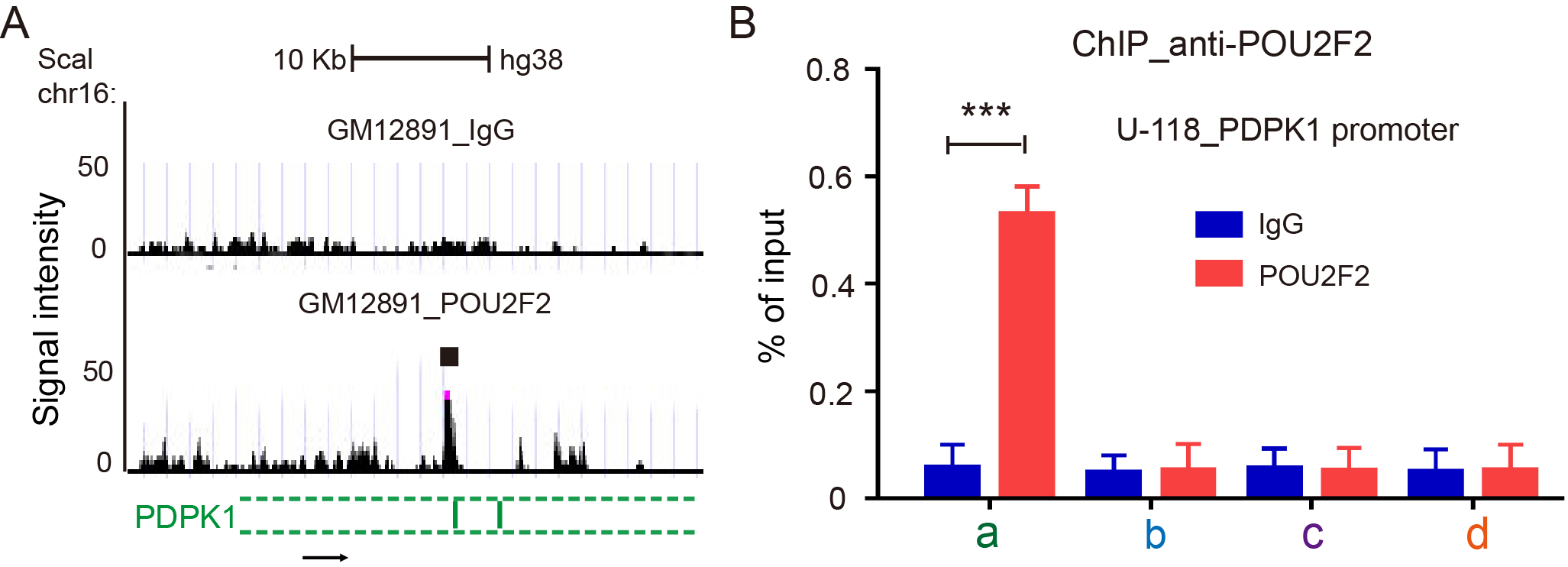

Supplement: Supplementary file 8 — Supplemental Figure S5 [file 41419_2021_3719_MOESM8_ESM.tif]

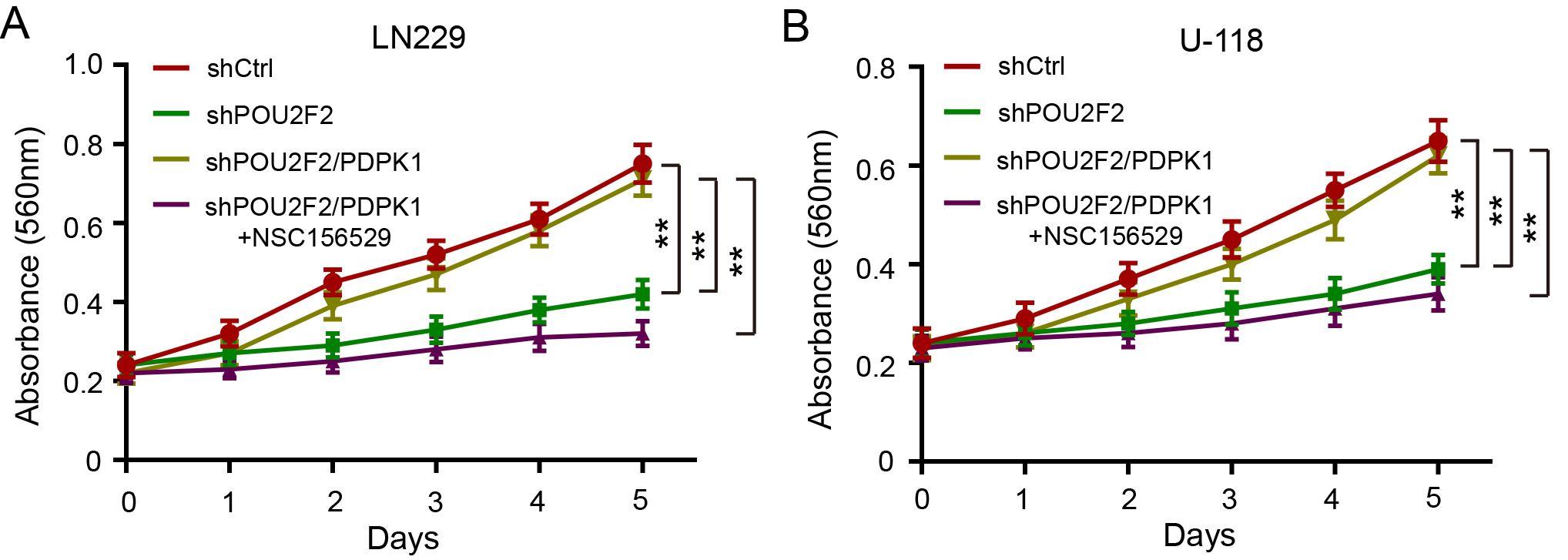

Supplement: Supplementary file 9 — Supplemental Figure S6 [file 41419_2021_3719_MOESM9_ESM.tif]
